# 5-Isopropyl­imidazolidine-2,4-dione monohydrate

**DOI:** 10.1107/S1600536812002838

**Published:** 2012-01-31

**Authors:** Alaa A.-M. Abdel-Aziz, Adel S. El-Azab, Abdulrahman M. Al-Obaid, Madhukar Hemamalini, Hoong-Kun Fun

**Affiliations:** a Department of Pharmaceutical Chemistry, College of Pharmacy, King Saud University, PO Box 2457, Riyadh 11451, Saudi Arabia; b X-ray Crystallography Unit, School of Physics, Universiti Sains Malaysia, 11800 USM, Penang, Malaysia

## Abstract

In the title compound, C_6_H_10_N_2_O_2_·H_2_O, the imidazole ring is essentially planar, with a maximum deviation of 0.012 (2) Å. In the crystal, mol­ecules are connected *via* N—H⋯O and O—H⋯O hydrogen bonds, forming a supra­molecular tape along the *a* axis.

## Related literature

For details and applications of hydantoins, see: El-Deeb *et al.* (2010 ▶); Rajic *et al.* (2006 ▶); Carmi *et al.* (2006 ▶); Sergent *et al.*, (2008 ▶); Yu *et al.* (2004 ▶). For related structues, see: Delgado *et al.* (2007 ▶); Ciechanowicz-Rutkowska *et al.* (1994 ▶). For the synthetic procedure, see: Abdel-Aziz (2007 ▶). For a description of the Cambridge Structural Database, see: Allen (2002 ▶).
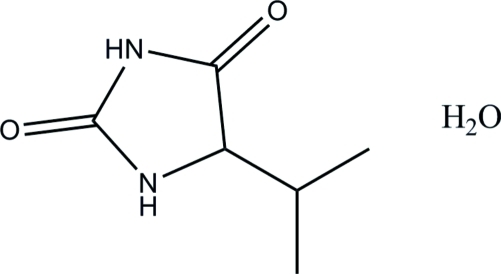


## Experimental

### 

#### Crystal data

C_6_H_10_N_2_O_2_·H_2_O
*M* *_r_* = 160.18
Orthorhombic, 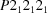

*a* = 6.2688 (3) Å
*b* = 9.2387 (4) Å
*c* = 14.8280 (7) Å
*V* = 858.77 (7) Å^3^
*Z* = 4
Cu *K*α radiation
μ = 0.84 mm^−1^
*T* = 296 K
0.90 × 0.21 × 0.16 mm

#### Data collection

Bruker SMART APEXII CCD area-detector diffractometer
Absorption correction: multi-scan (*SADABS*; Bruker, 2009 ▶) *T*_min_ = 0.518, *T*_max_ = 0.879
5702 measured reflections
1497 independent reflections
1378 reflections with *I* > 2σ(*I*)
*R*_int_ = 0.027

#### Refinement

*R*[*F* ^2^ > 2σ(*F* ^2^)] = 0.037
*wR*(*F* ^2^) = 0.098
*S* = 1.09
1497 reflections
117 parameters
H atoms treated by a mixture of independent and constrained refinement
Δρ_max_ = 0.12 e Å^−3^
Δρ_min_ = −0.18 e Å^−3^
Absolute structure: Flack (1983 ▶), with 592 Friedel pairs
Flack parameter: 0.2 (3)

### 

Data collection: *APEX2* (Bruker, 2009 ▶); cell refinement: *SAINT* (Bruker, 2009 ▶); data reduction: *SAINT*; program(s) used to solve structure: *SHELXTL* (Sheldrick, 2008 ▶); program(s) used to refine structure: *SHELXTL*; molecular graphics: *SHELXTL*; software used to prepare material for publication: *SHELXTL* and *PLATON* (Spek, 2009 ▶).

## Supplementary Material

Crystal structure: contains datablock(s) global, I. DOI: 10.1107/S1600536812002838/is5056sup1.cif

Structure factors: contains datablock(s) I. DOI: 10.1107/S1600536812002838/is5056Isup2.hkl

Supplementary material file. DOI: 10.1107/S1600536812002838/is5056Isup3.cml

Additional supplementary materials:  crystallographic information; 3D view; checkCIF report

## Figures and Tables

**Table 1:** Hydrogen-bond geometry (Å, °)

*D*—H⋯*A*	*D*—H	H⋯*A*	*D*⋯*A*	*D*—H⋯*A*
N1—H1N1⋯O2^i^	0.81 (2)	2.12 (2)	2.927 (2)	174.0 (19)
N2—H1N2⋯O1*W*^ii^	0.87 (3)	1.88 (3)	2.751 (2)	173 (2)
O1*W*—H1*W*1⋯O1	0.82 (4)	1.95 (4)	2.767 (2)	173 (3)
O1*W*—H2*W*2⋯O1^iii^	0.86 (4)	1.98 (4)	2.839 (2)	171 (4)

Symmetry codes: (i) 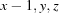; (ii) 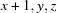; (iii) 
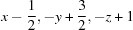
.

**Fig. 1.**
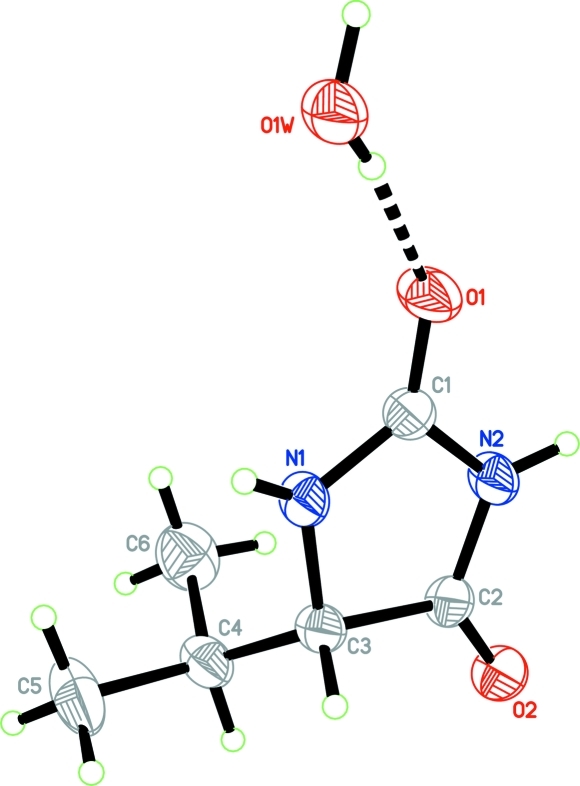
The asymmetric unit of the title compound, showing 50% probability displacement ellipsoids. The O—H···O hydrogen bond is shown by a dashed line.

**Fig. 2.**
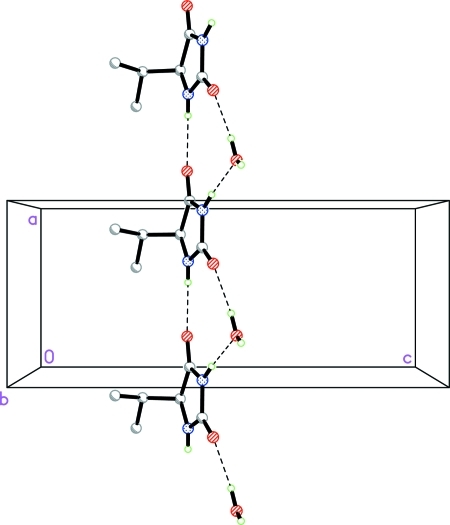
A partial packing view of the title compound. The hydrogen bonds are shown by dashed lines.

## supplementary crystallographic information

### Comment

Hydantoins (imidazolidine-2,4-dione) are important classes of compounds which have long attracted attention, owing to their remarkable biological and pharmacological properties, such as antitumor activity, antiviral activity, insulinotropic properties and EGFR inhibitors (El-Deeb *et al.*, 2010; Rajic *et al.*, 2006; Carmi *et al.*, 2006; Sergent *et al.*, 2008). The crystal structures of (*S*)-5-Benzylimidazolidine-2,4-dione monohydrate (Delgado *et al.*, 2007) and diphenylhydantoin derivatives (Ciechanowicz-Rutkowska *et al.*, 1994) have been reported in the literature. The title compound was successfully obtained in an optical active form without racemization by dehydrative cyclization in one-pot reaction of L-valine and urea in the presence of DPPOX as catalyst (Abdel-Aziz, 2007).

The asymmetric unit contains one (*S*)-5-isopropylimidazolidine-2,4-dione molecule and one water molecule as shown in Fig. 1. The imidazole (N1,N2/C1–C3) ring is essentially planar, with maximum deviations of 0.012 (2) Å for atom C3. The N1—C1—O1 [128.32 (19)°] angle is greater than the N2—C1—O1 [124.05 (17)°] angle. This difference is also observed in the hydantoin molecule (Yu *et al.*, 2004) and 50 other hydantoin derivatives reported in the Cambridge Structural Database (Version 5.28; Allen, 2002) with both unsubstituted NH groups and sp^3^-hybridization at C3. In the crystal structure (Fig. 2), the molecules are connected *via* N—H···O and O—H···O hydrogen bonds (Table 1), forming a supramolecular tape along the *a* axis.

### Experimental

The DPPOX (1.5 equiv) was added to the equimolar solution of L-valine and urea in MeCN in addition to Et_3_N (1.5 equiv) and the mixture was stirred at 50°C for 60 min. After removal of the solvent, the residue was taken up in organic solvent EtOAc, and washed successively with HCl aq and NaHCO_3_ aq. Evaporation of the dried organic solvent gave the title compound. The colourless single-crystals suitable for X-ray analysis was obtained by recrystallization from ethanol (m.p. 145–147 °C; yield: 95%).

### Refinement

Atoms H1N1, H1N2, H1W1 and H2W2 were located in a difference Fourier map and refined freely [N—H = 0.80 (2)–0.87 (3) Å; O—H = 0.82 (4)–0.87 (4) Å]. The remaining H atoms were positioned geometrically [C—H = 0.96 or 0.98 Å] and were refined using a riding model, with *U*_iso_(H) = 1.2 or 1.5*U*_eq_(C). A rotating group model was applied to the methyl groups. Even though there is sufficient anomalous dispersion to find the absolute configuration as the compound crystallize out in a chiral space group and Cu radiation was used, this was unsuccessful as the crystal is a inversion twin [BASF ratio of 0.8 (3):0.2 (3)].

### Crystal data

**Table:**

C_6_H_10_N_2_O_2_·H_2_O	*F*(000) = 344
*M**_r_* = 160.18	*D*_x_ = 1.239 Mg m^−^^3^
Orthorhombic, *P*2_1_2_1_2_1_	Cu *K*α radiation, λ = 1.54178 Å
Hall symbol: P 2ac 2ab	Cell parameters from 2281 reflections
*a* = 6.2688 (3) Å	θ = 5.6–65.9°
*b* = 9.2387 (4) Å	µ = 0.84 mm^−^^1^
*c* = 14.8280 (7) Å	*T* = 296 K
*V* = 858.77 (7) Å^3^	Needle, colourless
*Z* = 4	0.90 × 0.21 × 0.16 mm

### Data collection

**Table:**

Bruker SMART APEXII CCD area-detector diffractometer	1497 independent reflections
Radiation source: fine-focus sealed tube	1378 reflections with *I* > 2σ(*I*)
graphite	*R*_int_ = 0.027
φ and ω scans	θ_max_ = 67.3°, θ_min_ = 5.6°
Absorption correction: multi-scan (*SADABS*; Bruker, 2009)	*h* = −7→5
*T*_min_ = 0.518, *T*_max_ = 0.879	*k* = −11→10
5702 measured reflections	*l* = −17→17

### Refinement

**Table:**

Refinement on *F*^2^	Secondary atom site location: difference Fourier map
Least-squares matrix: full	Hydrogen site location: inferred from neighbouring sites
*R*[*F*^2^ > 2σ(*F*^2^)] = 0.037	H atoms treated by a mixture of independent and constrained refinement
*wR*(*F*^2^) = 0.098	*w* = 1/[σ^2^(*F*_o_^2^) + (0.0552*P*)^2^ + 0.0813*P*] where *P* = (*F*_o_^2^ + 2*F*_c_^2^)/3
*S* = 1.09	(Δ/σ)_max_ = 0.001
1497 reflections	Δρ_max_ = 0.12 e Å^−^^3^
117 parameters	Δρ_min_ = −0.18 e Å^−^^3^
0 restraints	Absolute structure: Flack (1983), 592 Friedel pairs
Primary atom site location: structure-invariant direct methods	Flack parameter: 0.2 (3)

### Special details

**Table:**

Experimental. ^1^H NMR (DMSO–d_6_): 10.54 (s, 1H, NH), 7.87 (s, 1H, NH), 3.89 (s, 1H), 2.01–1.97 (m, 1H), 0.94–0.92 (d, 3H, J = 7.0 Hz), 0.80–0.78 (d, 3H, J = 6.5 Hz). ^13^C NMR (DMSO–d_6_): 175.87, 158.28, 63.22, 30.01, 18.93, 16.31.
Geometry. All s.u.'s (except the s.u. in the dihedral angle between two l.s. planes) are estimated using the full covariance matrix. The cell s.u.'s are taken into account individually in the estimation of s.u.'s in distances, angles and torsion angles; correlations between s.u.'s in cell parameters are only used when they are defined by crystal symmetry. An approximate (isotropic) treatment of cell s.u.'s is used for estimating s.u.'s involving l.s. planes.
Refinement. Refinement of F^2^ against ALL reflections. The weighted R-factor wR and goodness of fit S are based on F^2^, conventional R-factors R are based on F, with F set to zero for negative F^2^. The threshold expression of F^2^ > 2σ(F^2^) is used only for calculating R-factors(gt) etc. and is not relevant to the choice of reflections for refinement. R-factors based on F^2^ are statistically about twice as large as those based on F, and R- factors based on ALL data will be even larger.

### Fractional atomic coordinates and isotropic or equivalent isotropic displacement parameters (Å^2^)

**Table:**

	*x*	*y*	*z*	*U*_iso_*/*U*_eq_	
O1	0.6583 (2)	0.56794 (15)	0.46328 (11)	0.0679 (5)	
O2	1.2150 (2)	0.27203 (15)	0.39487 (11)	0.0590 (4)	
N1	0.6703 (3)	0.34173 (17)	0.39871 (11)	0.0472 (4)	
N2	0.9719 (2)	0.44507 (16)	0.43611 (11)	0.0503 (4)	
C1	0.7524 (3)	0.46070 (19)	0.43523 (13)	0.0481 (4)	
C2	1.0337 (3)	0.31526 (19)	0.40226 (13)	0.0440 (4)	
C3	0.8324 (3)	0.23514 (18)	0.37618 (12)	0.0436 (4)	
H3A	0.8156	0.1507	0.4156	0.052*	
C4	0.8317 (3)	0.1851 (2)	0.27789 (14)	0.0534 (5)	
H4A	0.9533	0.1196	0.2701	0.064*	
C5	0.6312 (4)	0.0986 (3)	0.25796 (19)	0.0828 (8)	
H5A	0.6202	0.0197	0.2998	0.124*	
H5B	0.6381	0.0613	0.1976	0.124*	
H5C	0.5086	0.1602	0.2639	0.124*	
C6	0.8624 (5)	0.3102 (3)	0.21256 (16)	0.0810 (7)	
H6A	0.9926	0.3600	0.2266	0.122*	
H6B	0.7446	0.3760	0.2177	0.122*	
H6C	0.8694	0.2736	0.1520	0.122*	
H1N1	0.543 (4)	0.329 (2)	0.3981 (13)	0.045 (5)*	
H1N2	1.059 (5)	0.509 (3)	0.4591 (17)	0.078 (8)*	
O1W	0.2489 (3)	0.63212 (17)	0.52044 (13)	0.0665 (4)	
H1W1	0.374 (6)	0.617 (3)	0.507 (2)	0.096 (11)*	
H2W2	0.214 (6)	0.721 (4)	0.531 (3)	0.122 (13)*	

### Atomic displacement parameters (Å^2^)

**Table:**

	*U*^11^	*U*^22^	*U*^33^	*U*^12^	*U*^13^	*U*^23^
O1	0.0438 (9)	0.0566 (8)	0.1033 (12)	0.0068 (6)	−0.0017 (7)	−0.0302 (8)
O2	0.0292 (8)	0.0607 (8)	0.0871 (11)	0.0039 (6)	−0.0030 (6)	−0.0086 (7)
N1	0.0246 (8)	0.0507 (8)	0.0662 (10)	−0.0020 (6)	−0.0013 (6)	−0.0127 (7)
N2	0.0309 (9)	0.0475 (8)	0.0725 (11)	−0.0035 (6)	−0.0045 (7)	−0.0147 (8)
C1	0.0372 (10)	0.0474 (9)	0.0597 (12)	0.0028 (8)	−0.0024 (8)	−0.0079 (8)
C2	0.0312 (9)	0.0468 (9)	0.0541 (10)	−0.0018 (7)	−0.0011 (7)	−0.0001 (8)
C3	0.0323 (9)	0.0394 (8)	0.0591 (10)	−0.0025 (6)	0.0003 (7)	−0.0028 (7)
C4	0.0443 (11)	0.0491 (9)	0.0667 (12)	−0.0016 (8)	0.0028 (8)	−0.0161 (8)
C5	0.0645 (16)	0.0895 (17)	0.0945 (18)	−0.0206 (13)	−0.0049 (14)	−0.0376 (14)
C6	0.098 (2)	0.0838 (16)	0.0617 (14)	−0.0079 (15)	0.0041 (13)	−0.0044 (12)
O1W	0.0438 (10)	0.0557 (8)	0.1000 (12)	−0.0042 (7)	−0.0067 (8)	−0.0203 (8)

### Geometric parameters (Å, °)

**Table:**

O1—C1	1.226 (2)	C4—C5	1.518 (3)
O2—C2	1.210 (2)	C4—C6	1.520 (3)
N1—C1	1.329 (2)	C4—H4A	0.9800
N1—C3	1.454 (2)	C5—H5A	0.9600
N1—H1N1	0.80 (2)	C5—H5B	0.9600
N2—C2	1.357 (2)	C5—H5C	0.9600
N2—C1	1.383 (3)	C6—H6A	0.9600
N2—H1N2	0.87 (3)	C6—H6B	0.9600
C2—C3	1.513 (2)	C6—H6C	0.9600
C3—C4	1.529 (3)	O1W—H1W1	0.82 (4)
C3—H3A	0.9800	O1W—H2W2	0.87 (4)
			
C1—N1—C3	112.51 (15)	C5—C4—C3	110.30 (17)
C1—N1—H1N1	120.6 (15)	C6—C4—C3	112.16 (16)
C3—N1—H1N1	126.2 (15)	C5—C4—H4A	107.2
C2—N2—C1	111.89 (15)	C6—C4—H4A	107.2
C2—N2—H1N2	124.4 (19)	C3—C4—H4A	107.2
C1—N2—H1N2	123.6 (19)	C4—C5—H5A	109.5
O1—C1—N1	128.32 (19)	C4—C5—H5B	109.5
O1—C1—N2	124.05 (17)	H5A—C5—H5B	109.5
N1—C1—N2	107.63 (16)	C4—C5—H5C	109.5
O2—C2—N2	126.42 (17)	H5A—C5—H5C	109.5
O2—C2—C3	126.79 (16)	H5B—C5—H5C	109.5
N2—C2—C3	106.79 (15)	C4—C6—H6A	109.5
N1—C3—C2	101.12 (13)	C4—C6—H6B	109.5
N1—C3—C4	114.95 (15)	H6A—C6—H6B	109.5
C2—C3—C4	113.19 (15)	C4—C6—H6C	109.5
N1—C3—H3A	109.1	H6A—C6—H6C	109.5
C2—C3—H3A	109.1	H6B—C6—H6C	109.5
C4—C3—H3A	109.1	H1W1—O1W—H2W2	116 (3)
C5—C4—C6	112.4 (2)		

### Hydrogen-bond geometry (Å, °)

**Table:**

*D*—H···*A*	*D*—H	H···*A*	*D*···*A*	*D*—H···*A*
N1—H1N1···O2^i^	0.81 (2)	2.12 (2)	2.927 (2)	174.0 (19)
N2—H1N2···O1W^ii^	0.87 (3)	1.88 (3)	2.751 (2)	173 (2)
O1W—H1W1···O1	0.82 (4)	1.95 (4)	2.767 (2)	173 (3)
O1W—H2W2···O1^iii^	0.86 (4)	1.98 (4)	2.839 (2)	171 (4)

Symmetry codes: (i) *x*−1, *y*, *z*; (ii) *x*+1, *y*, *z*; (iii) *x*−1/2, −*y*+3/2, −*z*+1.
